# Adapt to Persist: Glioblastoma Microenvironment and Epigenetic Regulation on Cell Plasticity

**DOI:** 10.3390/biology11020313

**Published:** 2022-02-16

**Authors:** Daniel Uribe, Ignacio Niechi, Gorjana Rackov, José I. Erices, Rody San Martín, Claudia Quezada

**Affiliations:** 1 Institute of Biochemistry and Microbiology, Faculty of Sciences, Universidad Austral de Chile, Valdivia 5090000, Chile; uribebrange.d@gmail.com (D.U.); ignacio.niechi@uach.cl (I.N.); ignacioern@gmail.com (J.I.E.); rodysanmartin@uach.cl (R.S.M.); 2 Department of Immunology and Oncology, Centro Nacional de Biotecnología-Consejo Superior de Investigaciones Científicas (CNB-CSIC), 28049 Madrid, Spain; gorjanarackov@gmail.com; 3 Millennium Institute on Immunology and Immunotherapy, Universidad Austral de Chile, Valdivia 5090000, Chile

**Keywords:** glioblastoma stem-like cells, tumor niches, cell plasticity, GSCs subtypes, proneural–mesenchymal transition, epigenetic

## Abstract

**Simple Summary:**

Glioblastoma stem-like cells (GSCs) drive the progression and therapeutic resistance of glioblastoma. GSC plasticity allows them to adapt to different microenvironments and to persist after treatments. GSCs can reside in hypoxic, invasive and perivascular niches, which shape their phenotype through the induction of transitions involving metabolic and epigenetic changes. Therefore, the targeting of molecules that dynamically regulate the transcriptional programs of GSCs, and consequently their plasticity, has emerged as a novel therapeutic alternative. In this review, we described the intratumoral heterogeneity of GBM, discussing the role of GSCs niches and epigenetic modifications on the cell plasticity.

**Abstract:**

Glioblastoma (GBM) is the most frequent and aggressive brain tumor, characterized by great resistance to treatments, as well as inter- and intra-tumoral heterogeneity. GBM exhibits infiltration, vascularization and hypoxia-associated necrosis, characteristics that shape a unique microenvironment in which diverse cell types are integrated. A subpopulation of cells denominated GBM stem-like cells (GSCs) exhibits multipotency and self-renewal capacity. GSCs are considered the conductors of tumor progression due to their high tumorigenic capacity, enhanced proliferation, invasion and therapeutic resistance compared to non-GSCs cells. GSCs have been classified into two molecular subtypes: proneural and mesenchymal, the latter showing a more aggressive phenotype. Tumor microenvironment and therapy can induce a proneural-to-mesenchymal transition, as a mechanism of adaptation and resistance to treatments. In addition, GSCs can transition between quiescent and proliferative substates, allowing them to persist in different niches and adapt to different stages of tumor progression. Three niches have been described for GSCs: hypoxic/necrotic, invasive and perivascular, enhancing metabolic changes and cellular interactions shaping GSCs phenotype through metabolic changes and cellular interactions that favor their stemness. The phenotypic flexibility of GSCs to adapt to each niche is modulated by dynamic epigenetic modifications. Methylases, demethylases and histone deacetylase are deregulated in GSCs, allowing them to unlock transcriptional programs that are necessary for cell survival and plasticity. In this review, we described the effects of GSCs plasticity on GBM progression, discussing the role of GSCs niches on modulating their phenotype. Finally, we described epigenetic alterations in GSCs that are important for stemness, cell fate and therapeutic resistance.

## 1. Introduction

Glioblastoma (GBM) is the most frequent primary brain tumor accounting for more than 50% of gliomas in all age groups [1]. This tumor is characterized by the presence of poorly differentiated cells, nuclear atypia, high mitotic activity and foci of hypoxia-associated necrosis due to disorganized and inefficient tumor vasculature. GBM is refractory to multimodal therapy or Stupp protocol, consisting of radio and chemotherapy with temozolomide (TMZ) [2], and exhibits a 5-year survival of less than 5%, remaining as one of the most lethal neoplasias [3]. The efficacy of GBM therapies is further hampered by both inter- and intra-tumoral heterogeneity. Massive transcriptomic and genetic analyses identified three subtypes of GBM—classic, proneural (PN) and mesenchymal (Mes)—which present alterations in several signaling pathways and show different tumor growth patterns and response to therapies [4,5,6,7]. The PN subtype (PN-GBM) is mainly detected in secondary GBMs and in young patients (40.5 ± 1.4 years), while classic and Mes subtypes (Mes-GBM) are more frequent in older patients (49.0 ± 2.5 and 50.7 ± 1.3 years, respectively), showing both a primary and secondary origin [8,9]. PN-GBMs have been correlated to a better prognosis compared to the classic and Mes subtypes. Mes-GBM develops extensive tumor necrosis, inflammation, cell invasion and aberrant angiogenesis, which makes it the most aggressive GBM class with the worst prognosis [7,8,9,10,11,12].

GBM cells coexist with non-tumor cells that include endothelial cells, pericytes, astrocytes, neuronal precursor cells, fibroblasts and immune cells (microglia/macrophages, dendritic cells and leukocytes), which, together with components of the extracellular matrix, comprise the tumor microenvironment (TME). The interaction between different TME components creates specialized niches in which cancer cells acquire properties associated with tumor progression and resistance to treatment. Hypoxic and immune microenvironments are two examples of specialized TMEs, as they promote metabolic adaptation, multiple drug resistance and immunosuppression. Among the cellular components of TME, glioblastoma stem-like cells (GSCs) are among the most preponderant factors in GBM pathology [13]. Like neural stem cells (NSCs), GSCs have the ability to self-renew and differentiate into multiple lineages by activating transcriptional programs and signaling pathways that are typically activated during embryonic development. However, GSCs show a greater phenotypic plasticity and adaptability, enhanced tumorigenic properties, and elevated resistance to treatments compared to NSCs and non-stem cancer cells [14,15,16,17,18,19,20], so they have been considered an attractive therapeutic target. Tumor heterogeneity is also observed at a GSCs level. Transcriptomic, genetic and epigenetic analyses have identified two mutually exclusive subclasses of GSCs, PN-GSCs and Mes-GSCs, which are reminiscent of the PN and Mes clinical phenotypes described for GBM. Mes-GSCs show a more radioresistant phenotype and have been associated with a worse prognosis than PN-GSCs [21,22]. It has been well documented that PN-to-Mes transition (PMT) can be induced by therapy and is therefore recognized as a treatment resistance mechanism and metabolic adaptation [21,22,23]. GSCs exhibit a flexible transcriptional program that allows them to transit between different cellular substates with distinctive phenotypic and functional characteristics [24,25]. Depending on their niche or external factors (such as therapy), GSCs can express a quiescent, proliferative or invasive phenotype or become differentiated cancer cells. GSCs reside in different tumor niches, which not only ensures their maintenance but also provides signals to adapt, progress and promote treatment resistance [26,27,28,29]. Three niches have been described for GSCs: hypoxic/necrotic, invasive and perivascular. The hypoxic/necrotic niche induces metabolic changes allowing GSCs to survive in nutrient-restricted conditions and adopt a quiescent phenotype, resistant to treatments and with great potential to infiltrate healthy tissue. The invasive niche or invasion edge encourages GSCs to acquire a mesenchymal phenotype, which favors cell migration and invasion through the expression and activity of proteins that mediate the epithelial–mesenchymal transition (EMT). The perivascular niche harbors GSCs that are closely associated with endothelial cells and pericytes. This association promotes GSCs self-renewal, which in turn remodels the tumor vasculature through transdifferentiation and delivery of regulatory molecules [30,31,32,33,34,35].

The GSC ability to adapt to different niches implies that these cells dynamically restructure their transcriptional program, inducing the transient expression of genes with specific functions for each cell state [18,36]. In this context, epigenetic modifications are an efficient mechanism for GSCs, since these are reversible events that do not affect DNA primary sequence, allowing them to access a spectrum of genes quickly and efficiently [37]. DNA and histone methylation/demethylation are two widely studied phenomena in gliomas [38,39,40,41]. Methylases and demethylases are deregulated in GSCs, resulting in a different, less restricted and more unpredictable epigenetic landscape than non-GSCs cells. However, these modifications seem not to be random, since they ensure stemness of the GSCs, regulate their cell fate and resistance to treatments. Therefore, targeting the epigenetic machinery as emerged as an attractive alternative to counteract GSCs plasticity and to impair tumor heterogeneity [42,43]. This review addresses the role of GSCs in tumor progression from the perspective of their cellular plasticity and how their different niches shape their phenotype and ensure their survival. Furthermore, we will describe general aspects of epigenetic modifications in GSCs, emphasizing genome methylation and their role in regulating the stem-like phenotype.

## 2. Glioblastoma Stem-like Cells, Subtypes and Cellular Plasticity

The biological complexity of GBM was revealed when Ignatova and colleagues first identified a cellular subpopulation with stem cell properties in brain tumors [44]. These cells were later called GSCs, and like NSCs, they have the ability to self-renew and differentiate into multiple cell lineages [14,16,45,46] However, GSCs continuously proliferate, rise genetic variability, generate progeny with different grades of differentiation and possess a high potential to initiate a tumor when xenotransplanted into an immunodeficient mice brain [45]. GSCs are resistant to both chemo- and radiotherapy, even more so than bulk tumor cell populations [14,47]. In addition, it is known that GSCs exhibit elevated migratory and invasive potential, eliciting tumoral infiltration into healthy tissue, which prevents the total surgical resection of the tumor mass and limits the localized effect of radiotherapy [48]. Residual cells have the ability to regenerate GBM in brain regions distant from the initial tumor by acquiring new and different driver mutations. In low-grade gliomas or GBMs with IDH-wild-type, treatment with TMZ can even promote the appearance of these mutations, thereby limiting treatment options [49].

This bleak landscape is even more complex if we consider that GBM is molecularly heterogeneous at the inter-patient level, which implies challenges in diagnosis, monitoring and treatment. GBM heterogeneity is also interpolated to GSCs: two subtypes of GSCs have been identified by differential gene expression, proneural (PN-GSCs) and mesenchymal (Mes-GSCs), which resembles the genotype of their parental tumors. Mes-GSCs express CD44, YKL40 (CHI3L1), Lyn, WT1 and BCL2A1, show a more proliferative phenotype in vitro and in vivo, more radioresistance and have been associated with a worse prognosis than PN-GSCs [21]. Additionally, Mes-GSCs develop tumors with abundant blood vessels, hemorrhagic lesions and extensive necrotic areas. In fact, Mes-GSCs are enriched with genes related to angiogenesis, inflammation, cell migration/invasion and activation of metabolic pathways mediated by glycolysis and NF-κB. PN-GSCs express CD133, EZH2, Olig2, Sox2 and Notch1, are enriched with genes that participate in the regulation of glial and neural homeostasis, cell cycle, DNA repair and activation of Notch and PDGF receptor signaling pathways. In addition, PN-GSCs exhibit a less proliferative phenotype in vitro and in vivo than Mes-GSCs, which has been associated with a better prognosis [21,22,50]. Recently, we have demonstrated that Mes-GSCs exhibit higher extracellular adenosine levels than PN-GSCs [51], which is remarkable considering that signaling is aberrantly activated under hypoxia, thereby promoting chemoresistance and cell invasion [52,53,54]. Altogether, these data suggest that adenosine signaling enhances mesenchymal traits on GBM cells.

While early studies suggested that PN-GSCs and Mes-GSCs would be mutually exclusive within the tumor [21,50,55], evidence shows that both subtypes can coexist through dynamic transitions [56]. A transition from a PN to a Mes (PMT) phenotype can be induced by TMZ treatment, TNF-α or radiation by an NF-κB-dependent mechanism [21,22,50]. These data suggest that the presence of PN-GSCs or Mes-GSCs within the tumor would respond to an adaptive enrichment of a particular subtype that is promoted by therapy and changes of tumor microenvironment. A study that addressed both aspects showed that the combined treatment of TMZ with radiation enriched CD44+ cells, while reducing the population of CD133+ cells. In addition, authors demonstrated that under hypoxic conditions, there was an enrichment of the CD133+/CD44-population and a decrease in the CD44+/CD133-population [57]. The intratumoral distribution of PN-GSCs and Mes-GSCs also seems to be different, depending on microenvironment. Jin and colleagues observed in tumor tissue samples that GSCs with PN signal (Sox2+ and Olig2+) were located in perivascular niches, while GSCs with Mes signal (CD44+ and YKL40+) occupied exclusively hypoxic/necrotic regions. The areas between the necrotic and perivascular region showed a mixture of PN and Mes markers, implying that both GSC subtypes coexist in the same tumor [58].

Growing evidence has challenged the hierarchical model of cancer, which positions cancer stem cells (CSCs) at the apex of a hierarchical cellular organization. This model explains tumor progression as a unidirectional process that begins with CSCs and evolves into more differentiated cell progeny, enhancing intratumoral heterogeneity. However, new studies suggest that intratumoral heterogeneity is the result of the plasticity of GSCs rather than their multi-potency. Dirkse et al. observed that stem markers such as CD133, CD44, CD15 and A2B5 are heterogeneously distributed in GBM tissue, which is also observed at the single spheroid level. Based on the combined expression of the 4 stem markers, authors isolated 16 GSC subpopulations and demonstrated that any of these has the potential to reconstitute all other subpopulations with their respective markers [59]. These data suggest that GSCs can transit through different phenotypic states, not exclusively leading to unipotently differentiated progeny as suggested by the hierarchical model. The model of cellular states, on the other hand, integrates these observations, postulating that the GBM contains cells in a limited set of phenotypic states that resemble the (i) neural progenitor-like, (ii) oligodendrocyte-progenitor-like, (iii) astrocyte-like and (iv) mesenchymal-like states, and that each cell has the potential to generate any of the four states [24]. The frequency of these states can vary between different GBMs due to specific genetic alterations that favor the prevalence of one or another state, which could explain, for example, the differences in the speed of tumor progression between different patients.

Transcriptomic analyses have suggested that GSCs are in a state of high entropy, which translates into a flexible transcriptional program allowing them to make transitions from one phenotypic state to another [56,60,61]. This phenomenon would ensure GSCs adaptation to different tumor niches, expanding the options of therapeutic resistance mechanisms. In contrast, differentiated cancer cells are in a low entropy state, with a reduced and strictly regulated transcriptional program, which would restrict their cell plasticity and adaptability [34,56,60,61,62]. It has been postulated that the transitions of GBM cells between one state and another are influenced by a series of intrinsic and extrinsic “attractors” to the tumor. The attractor state model postulates that attractors such as the cell niche, genetic alterations and/or therapy influence the change of a cell state and that GSCs would have a greater capacity for response and adaptation than their differentiated counterpart due to their greater transcriptional capacity and flexibility [62]. Therefore, understanding how attractors shape the cellular phenotype could help us avoid the adaptation, heterogeneity and progression of GBM cells.

## 3. GSCs Niches

In the adult mammalian brain, NSCs reside in two regions, the ventricular–subventricular zone and the subgranular zone (V-SVG and SVZ). NSCs are maintained by a permissive microenvironment, where their direct or indirect interaction with ependymal cells, endothelial cells, microglia, progenitor cells as well as with soluble factors from blood and cerebrospinal fluid, promote their self-renewal and regulate their proliferation and cell differentiation. [63,64,65]. Similarly, the microenvironment of GSCs also modulates their phenotype, functioning not only as a regulator of their stem characteristics but also as a promoter of their resistance to treatments [66]. At least three major microenvironments have been recognized for GSCs, (1) the peri-necrotic/hypoxic niche, (2) the perivascular niche and (3) the invasion edge, all of them with the ability to shape the phenotype of GSCs and ensure their heterogeneity [13].

### 3.1. Hypoxic Niche

GBM is a highly vascularized tumor but exhibits extensive hypoxic areas associated with necrosis. This paradoxical phenomenon is explained by structural and functional abnormalities of blood vessel formation in response to fast tumor growth [67]. Hypoxia has been strongly linked to GBM progression and chemoresistance, contributing to therapeutic failure and poor prognosis [68,69]. It has been reported that oxygen levels in the brain oscillate between 12.5% and 2.5% (physioxia), but in tumor tissue, oxygen levels decrease at 2.4% or 0.1% (hypoxia) [70,71,72]. The ability of cells to adapt to this hypoxic microenvironment is regulated by a family of transcription factors denominated Hypoxia-Inducible Factors (HIFs), with HIF-1α and HIF-2α being the most studied [73]. Although HIF-1α and HIF-2α may appear redundant with respect to their target genes, the difference is in the timing of their response to low oxygen levels [74]. HIF-1α mediates the cellular response to acute hypoxia, reaching its protein peak between 4 and 8 h, while HIF-2α peaks later on, between 24 and 72 h, mediating the cellular response to chronic hypoxia [74]. However, the transcriptional activity of both HIFs has been associated with the regulation of key aspects of GBM such as clonogenicity, metabolic adaptation, angiogenesis, chemoresistance and migration/invasion [70,75,76,77,78].

The hypoxic zones associated with necrosis within GBM, such as the pseudopalisades, are enriched with GSCs [79]. As evidenced in NSCs, hypoxia promotes self-renewal and inhibits differentiation of GSCs, whereas in differentiated cancer cells (non-GSCs) hypoxia induces stem characteristics such as neurospheres formation, self-renewal and stem marker expression [30,80,81,82,83,84,85,86]. In addition to promoting the stem phenotype, hypoxia promotes the enrichment of GSC subpopulations with distinctive phenotypic and functional characteristics, allowing them to adapt to the hypoxic niche. The question is whether the GSC enrichment under hypoxia is a result of the hypoxia-resistant cell selection or cell adaptation. A study showed that different GSC subpopulations self-renew and proliferate indefinitely under hypoxia. Although some subpopulations had a lower capacity for self-renewal in the initial passages, all cell cultures achieved to survive and reach the same equilibrium under hypoxia, which was characterized by a heterogeneous expression of the markers CD133, CD44, CD15 and A2B5 [59]. These data suggest that GSCs are not selected based on a resistant phenotype but rather adaptation to hypoxia. Low oxygen levels induce a shift from a proliferative state to a quiescent state in GSCs, which is characterized by a reversible cell arrest in G0 [27,79,87]. In the quiescent state, it decreases the expression of genes associated with cell cycle progression such as CCNA2, CCNB1 and CCNE2 and increases the expression of cycle inhibitors such as CDKN1A and G0S2 [88,89,90]. In a study where quiescent GSCs (qGSCs) and proliferative GSCs (pGSCs) were isolated from GBM organoids, it was demonstrated that qGSCs exhibit high expression of hypoxia-associated genes, such as those that code for NF-kB, TNF-α and the IL6/JAK/Stat3 axis in relation to pGSCs [27]. Quiescent GSCs have posed a challenge for research and therapy as, being in a state of latency, these cells escape antitumor surveillance systems and become refractory to treatments that target rapidly proliferating cells [28,91]. In fact, qGSCs are also more resistant to radiotherapy and chemotherapy with TMZ [27]. Furthermore, qGSCs are present in the tumor before therapy and are enriched after treatment with TMZ or RTK inhibitors, which allows them to drive tumor growth as a prelude to GBM recurrence [26,36]. Interestingly, pGSCs and qGSCs exhibit similar characteristics to PN-GSCs and Mes-GSCs subtypes, respectively. For example, pGSCs and PN-GSCs are often found in vascularized niches, are more sensitive to therapy than their Mes counterparts and are associated with a better clinical prognosis. In contrast, qGSCs and Mes-GSCs inhabit hypoxic and invasive niches, are more resistant to therapy and are associated with patient poor prognosis [21,26,27,36,79,92]. Similar to radiochemotherapy-induced PMT, pGSCs can change to qGSCs after exposure to adverse factors such as hypoxia, acidification and radiochemotherapy [21,22,27,93,94]. However, it is not clear whether the proliferative and quiescent states correspond to the PN and MES molecular subtypes or to transitions that can be generated in either of the two GSC subtypes. In fact, Mes-GSCs show fast proliferation in vitro, so their phenotype does not necessarily have to be related to a quiescent state.

Together, these studies demonstrate that GSCs persist under hypoxic microenvironments due to dynamic phenotypic transitions, which are characterized by metabolic changes associated with therapeutic resistance. These changes allow GSCs to reach a cellular state with intrinsic adaptive capacity, which ensures their survival under hypoxia and their conjugation with other niches to promote tumor progression.

### 3.2. Invasive Niche

The border that delimits the tumor from healthy tissue represents a specific microenvironment that constitutes another niche for GSCs. These cells have a high capacity for migration/invasion into the surrounding healthy tissue, which is why they have been attributed a direct responsibility for the appearance of new tumor foci after surgical intervention and the imminent tumor recurrence [49,95,96]. Migration and invasion processes require a phenotypic change of the GSCs reminiscent of the epithelial–mesenchymal transition (EMT). GBM is not an epithelial tumor, so the term EMT is not applicable to this cancer. Instead, PMT might be a more appropriate concept for the brain context [6,21,22,97]. Immunohistochemical analysis of GBM sections obtained from the core and periphery of the tumor, as well as GSCs isolated from these two regions, showed that the invasion edge expresses high levels of the CD133 and Olig2 PN markers relative to the tumor core. In contrast, the CD44 Mes marker is poorly expressed in the periphery but is enriched in the innermost region of the tumor. Importantly, data from this study demonstrated that radiotherapy induces PMT in GSCs isolated from the periphery, suggesting that this type of therapy can enrich a more aggressive cellular phenotype and contribute to the appearance of new tumor foci [93]. About 15 years ago, when GSCs were just beginning to be isolated and their phenotypic subclasses were not yet described, Farin et al. demonstrated that GBM cells intermittently migrate within the tumor, pausing for one hour to divide before reestablishing their migration [98]. This “proliferation on route” may involve transitions between GSC proliferative-invasive states or PMTs, such that the enrichment of a marker in a specific tumor region could be the consequence of these transitions rather than its intrinsic association with that niche.

The shift of GSCs toward a more invasive phenotype involves the induction of a series of EMT-type transition modulators, such as E-cadherin (with the concomitant decrease in N-cadherin), the Twist-Sox2 axis, Snail, ZEB, STAT3, periostin and NF-kB [99,100,101,102,103]. Through integrins and cadherins, GSCs migrate through the brain parenchyma, disassembling the extracellular matrix due to the activity of matrix metalloproteinases, such as MMP2, MMP9 and ADAMT2, and invading along the ventricular and vascular basement membranes [104,105]. GSC migration and invasion can be promoted by soluble factors from the tumor microenvironment, such as transforming growth factor beta 1 (TGF-β1) and adenosine. TGF-β1 is secreted by microglia and activates the TGF-β2 receptor (TGFRβ2) in GSCs and increases cell invasion [106]. Through autocrine signaling, TGF-β also regulates EMT/migration/invasion processes in GSCs through the AXL/EZH2/TGF-β1 axis or through hypoxia-dependent transcriptional activity of ZEB1 [103,107,108]. On the other hand, hypoxia promotes the extracellular adenosine production in GSCs, which positively regulates cell migration and invasion through the adenosine A3 receptor [53]. The effects of this nucleoside on migration and invasion can be diminished using recombinant adenosine deaminase in order to promotes extracellular adenosine depletion [54].

The perivascular niche has also been shown to regulate cell migration/invasion. For example, the pro-angiogenic protein Angiopoietin-1 (Ang-1) promotes the adhesion of GSCs to endothelial cells and increases in vitro cell invasion [109]. The effect of endothelial cells on GSCs invasion was also observed by McCoy and colleagues, who used 3D co-culture systems with GSCs and human brain microvascular endothelial cells and demonstrated increased cell invasion and interleukin-8 (IL-8)-dependent stem marker expression in relation to monocultures of GSCs [33]. These data suggest that endothelial cells not only promote the migration/invasion of GSCs but also ensure their stem phenotype during these processes. Another important aspect to highlight is that cell invasion and angiogenesis in GBM appear to be temporally exclusive events. In vivo studies showed that MMP2 knockout (KO) developed a higher blood vessel density in the tumor, which was correlated with higher expression of the vascular endothelial growth factor receptor 2 (VEGFR2) compared to wild type [110]. Lu and colleagues demonstrated that GBM cells cultured with VEGF decrease their migratory capacity through a mechanism involving MET-VEGFR2 interaction, while VEGF KO cells exhibit increased expression of EMT markers [111]. By MRI analysis of GBM patients, Nishikawa et al. observed that highly invasive tumors were correlated with low VEGF expression in the tumor periphery, an area with high expression of CD44 associated with the invasive phenotype of GSCs [112]. In fact, anti-VEGF therapies such as bevacizumab have proven transient effects and may even cause a change in phenotype toward a predominantly infiltrative pattern in patients with GBM [113,114,115]. Furthermore, the activation of alternative angiogenic pathways mediated by the hypoxic microenvironment has been proposed as one of the resistance mechanisms, underlining the influence of tumor niches on the adaptability and consequent heterogeneity of GSCs [116,117].

### 3.3. Perivascular Niche

GBM perivascular microenvironment comprises a collaborative association between cancer cells, endothelial cells, pericytes, astrocytes and tumor-associated macrophages (TAMs), which form a niche that preserves GSC stem characteristics [13]. Endothelial cells provide ligands and secrete endogenous modulators that activate GSCs signaling, such as Notch, Sonic Hedgehog and nitric oxide [118,119,120,121,122]. On the other hand, GSCs produce pro-angiogenic factors such as VEGF, which signals through VEGFR2 expressed in endothelial cells, promoting cell migration toward the tumor parenchyma and, consequently, angiogenesis [83,123,124,125]. In turn, endothelial cells also produce VEGF and promote the proliferation of GSCs through VEGFR2 [126]. GSCs express an adhesion protein L1CAM that, through its interaction with αvβ3 integrin expressed in the vascular endothelium, promotes GSC stem phenotype and supports endothelial cell migration processes [127,128]. Furthermore, the GBM vasculature is also composed of tubular networks formed by glioma cells called tumor microtubes (TMs). TMs function as intercommunicating pathways between distant cells, supporting proliferation, invasion and resistance to treatments [35,129]. One study showed that the radio and chemoresistance of GBM cells associated with the perivascular niche and TMs is dependent on the activation of Notch1. The knockdown of this gene decreases vascular co-option and reduces the perivascular cell population but induces the formation and elongation of TMs [35]. This collateral effect not only demonstrates that a tumor niche can compensate for the decline of another niche but also underlines the importance of the phenotypic adaptation of the cells that compose it.

Additionally, GSCs can remodel the GBM vasculature by transdifferentiation to endothelial cells and pericytes, through mechanisms involving the transcription factor ETV2 and TGF-β, respectively [130,131,132]. Studies have shown that the transdifferentiation of GSCs to endothelial cells can be induced by both TMZ chemotherapy and radiotherapy [131,132]. Together, these studies underscore the importance of understanding the mechanisms that regulate the plasticity of GSCs, both to search for new therapeutic targets and to help predict tumor response to treatments.

## 4. Epigenetic Aspects of the GSCs Plasticity

The adaptation of GSCs to different tumor microenvironments requires a permissive transcriptional program that allows them to transit between different cell states. In this context, epigenetic modifications play a fundamental role since, unlike genetic alterations, the former can be reversible, allowing the cellular phenotype to be temporarily shaped based on the activation or repression of various genes. Epigenetics addresses those heritable alterations that do not involve changes in the DNA sequence and that are the consequence of different mechanisms such as histone remodeling and modification, DNA methylation, regulation by polycomb group proteins and regulation by microRNAs [18,37].

As in NSCs, epigenetic modifications in GSCs are important for the regulation of cell potency and fate (Table 1). DNA methylation is catalyzed by enzymes called DNAs methyl transferases (DNMTs), such as DNMT1, DNMT3A and DNMT3B, which transfer a methyl group from S-adenosyl-L-methionine to the C5 position of cytosine residues in DNA, forming 5-methylcytosine (5mC) [133,134]. On the other hand, the ten eleven translocation proteins (TET1, TET2 and TET3) catalyze the oxidation of 5 mC to 5-hydroxymethylcytosine (5hmC), 5-formylcytosine (5 fC) and 5-carboxycytosine (5 caC) in a process that leads to DNA demethylation [135,136,137]. In GSCs, many of these enzymes are deregulated in relation to NSCs, which has been associated with resistance to treatments and tumor heterogeneity. In a study where the transcriptomic and epigenetic profile of fetal brain-derived NSCs was compared with GSCs obtained from GBM patient-derived xenografts, it was shown that the latter have deregulated expression of TETs, associated with an increase in 5 fC and 5 caC modifications at the expense of a substantial loss of 5 mC and 5 hmC marks. These alterations were in turn correlated to an increase in chemoresistance, probably due to a greater expression of DNA repair genes in GSCs in relation to NSCs. This study further demonstrated that, under conditions of cell differentiation, GSCs exhibit an altered subcellular localization pattern for TET3. Specifically, in a subset of GSCs, TET3 tended to remain in the nucleus (TET1 and TET2 translocated to the cytoplasm) and prevented the expression of GFAP, suggesting that the redistribution of this enzyme could partly explain the heterogeneity of the cell lineage observed after GSC differentiation [20]. Additionally, the GSCs stemness is promoted by the action of DNMTs. The Sox2 and Oct4 gene reprogramming factors bind and transactivate promoters from DNMTs, which globally methylate DNA. This methylation results in the inhibition of miRNA-148a expression, which counteracts the stem phenotype [138].

Histone modification is another process that has been linked to the regulation of stem phenotype and GSC differentiation. GSCs exhibit a more dynamic and decondensed chromatin organization compared to differentiated cancer cells, which has been associated with a more flexible transcriptional program [139]. Histones are methylated by polycomb group proteins, promoting chromatin compaction and gene silencing. The term polycomb was initially conferred on a Drosophila mutant showing inadequate body segmentation. Currently, the polycomb group (PcG) refers to a group of genes whose mutations cause a phenotype similar to polycomb. PcG proteins are found in a variety of multiprotein complexes, including polycomb repressive complexes 1 and 2 (PRC1 and PRC2) [140]. PRC2 is the most widely characterized complex, and it is composed of four different proteins called Ezh1/2, Suz12, Eed and RbAp46/48. Ezh1/2 is the functional component that catalyzes the addition of methyl groups to lysine 27 on histone H3 (H3K27), forming di- or tri-methylated H3K27 (H3K27me2/me3) [141]. On the other hand, demethylation processes are carried out by histone demethylases, which can be divided into two families: amino oxidase homolog lysine demethylases (KDMs) and JmjC domain-containing histone demethylases [142]. H3K27me3 mediated by PRC2 has been shown to play a fundamental role in the plasticity of GSCs, specifically on the interconversion to differentiated tumor cells. After induction of differentiation in GSCs, the Ezh2-mediated H3K27me3 modification is enriched on Nanog and BMP5 promoters (genes associated with stemness), repressing their expression. In contrast, H3K27me3 is lost in the Wnt1 promoter, suggesting a switch between these genes during the interconversion of GSCs to differentiated tumor cells [143]. Interestingly, by analysis of GBM specimens, it was shown that the majority of cells with nuclear Ezh2 were found around tumor vessels and on the invasion front, while cytoplasmic Ezh2 was enriched in tumor core cells. These data suggested that Ezh2 could regulate cell differentiation processes in response to signals from the microenvironment [143]. Furthermore, the H3K27me3 modification has been shown to be necessary for the slow-cycling or quiescent state of GSCs. Liau and colleagues demonstrated that antitumor drug-induced transition from proliferative GSCs to slow-cycling GSCs is accompanied by extensive repressive redistribution of H3K27me3 and upregulation of KDM6, a H3K27me3 demethylase. Authors suggested that this demethylation could allow the activation of alternative cis regulatory elements to support the activation of genes that are necessary for cellular adaptation [36]. Another study demonstrated that KDM2B, a demethylase member of the JmjC family that removes methyl groups from H3K36me2 and H3K4me3, is enriched in GSCs compared to their differentiated counterparts. The downregulation of KDM2B reduced the population of GSCs and sensitized them to chemotherapy [144]. These antecedents suggest that demethylase activity in GSCs could be linked to the maintenance of the stem phenotype and to the regulation of cell differentiation. In fact, the knockdown of two other demethylases, KDM4C and KDM7A, induces cellular differentiation and DNA damage in GSCs [139].

Other widely studied histone modifications in cancer are acetylations, which are antagonistically regulated by histone acetyltransferases (HATs) and histone deacetylases (HDACs). HATs add acetyl groups on histone lysine residues, which neutralizes their positive charges and weakens their interaction with DNA, generally facilitating gene expression. HDACs not only remove acetyl groups from histones but also interact with transcription factors, acting as co-repressors or co-activators of gene expression [145]. HDACs have been widely studied in GBM cells due to their relationship with therapeutic resistance, cell proliferation and invasion, angiogenesis and apoptosis [146,147,148,149]. In fact, a wide variety of HDAC inhibitors (HDACi) have been tested in clinical trials for the treatment of different types of cancer, including GBM [150,151]. In GSCs, induction of KLF9 transcription factor expression in the presence of HDACi panobinostat negatively affects cell cycle and induces apoptosis and necroptosis [152], while treatment with the HDACi TSA and MS-275 reduces neurosphere growth leading to cell differentiation and apoptosis [153]. In particular, the function of HDACs has been strongly associated with the regulation of GSC stemness and senescence. Senescence corresponds to a terminal cellular state in which cells arrest their growth and stop cell division. In the context of tumor, senescent cancer cells decrease their tumorigenic potential and are more susceptible to therapy [154]. Normally, somatic cells have a limited proliferation capacity due to the shortening of telomeres (repetitive protective sequences located at the ends of chromosomes). In cancer cells, telomere attrition is partially prevented thanks to the addition of telomeric repeats by the enzyme telomerase, whose catalytic core is composed of a reverse transcriptase (TERT) and an RNA template [155]. Mutations in the TERT promoter are found in 60–80% of GBMs and have been associated with increased telomerase activity (TA). HDACs have been shown to downregulate TERT transcript levels, while increasing its protein stability [156]. The HDAC1/2/6/Sp1 pathway upregulates TERT, and treatment with azaindolyl sulfonamide (MPT0B291), an inhibitor of HDAC6 with partial effect on HDAC1/2, induces G2/M arrest and senescence in GSCs [157], as well as decreased growth in both TMZ-sensitive and -resistant cells [149]. Data suggest that TA is increased in GSCs relative to differentiated glioma cells. Serum-induced differentiation of GSCs downregulates telomerase and shortens telomeres, thereby inducing GSC senescence [158]. To maintain telomere integrity, GSCs with low or no TA use another mechanism called alternative lengthening of telomeres (ALTs), based on homologous recombination [159]. It has been shown that GSCs exhibiting ALTs are more resistant to ionizing radiation than those with TA phenotype [160]. On the other hand, knockdown of MUC1, a transmembrane protein associated with GBM progression, downregulates TERT causing a shift from TA to ALTs phenotype, suggesting that the activation of telomere maintenance mechanisms is compensatory rather than exclusionary [161].

Taken together, all these data indicate that the reorganization of chromatin in GSCs is crucial to stemness maintenance, to acquire competent cell states and to regulate cell differentiation. Since the epigenetic profiles and the dynamics of their changes are different between GSCs and non-GSCs (including NSCs), regulatory components of chromatin can be considered as attractive therapeutic targets to counteract the plasticity of GSCs and tumor heterogeneity.

**Table 1:** Epigenetic modifications and its role in regulating the GSCs plasticity.

Epigenetic Modification	Epigenetic Regulators	Biological Effect on GSCs	Reference
(↑) H3K27me3 on the *Nanog* promoter	EZH2	Inhibition of cell differentiation	[146]
(↑) Repressive methylation of miRNA-148a	*DNMT1, DNMT3b*	GSCs maintenance	[141]
- (↑) Active H3K27ac on the *WNT5A* and *DLX5* promoters - (↓) Repressive H3K27me3 on the *WNT5A* and *DLX5* promoters - (↑) Repressive H3K27me3 on the *PAX6* promoter	Not described by study	GSCs enrichment and endothelial differentiation	[19]
(↓) Repressive H3K27me3 on the *HEY1* and *HES5* promoters	KDM6A/B	Maintenance of slow-cycling GSCs	[36]
(↑) Active H3K27ac on the *HEY1* and *HES5* promoters	Not described by study		
- (↓) 5mC - (↑) 5fC/5caC	TET2	Promotes DNA repair genes/chemoresistance	[20]
	Nuclear TET3	Inhibition of cell differentiation	
(↓) H3K36me2	KDM2B	GSCs maintenance and chemoresistance	[144]
(↓) H3K9me3	KDM4C and KDM7A	GSCs maintenance and DNAdamage repair	[139]
(↑) H3K9ac	Not described by study		
(↓) Repressive methylation on the *Irf8*, *Nt5e* and *Cd274* promoters	Not described by study	Immune evasion	[162]
Sp1 deacetylation	HDAC6	Cell cycle progression and inhibition senescence	[149,157]
Not described by study	HDAC	Vasculogenic mimicry	[148]
Not described by study	HDAC	Prevention of apoptosis, necroptosis and cell cycle	[152]
Not described by study	HDAC	Prevention of apoptosis and cell differentiation	[153]
Not described by study	HDAC	Cell proliferation and prevention of cell differentiation	[163]

(↑) or (↓) indicate increase or decrease of epigenetic modification, respectively.

## 5. Conclusions and Future Prospects

GSCs have been grouped into two molecular subtypes, PN and Mes, each with different implications for tumor development and response to treatments. Although the use of personalized therapy has been proposed based on the prior identification of the molecular subtype, studies have shown that GSCs can shift between one subtype and another. Furthermore, quiescent and proliferative cellular substates could be expressed in each subtype, making it difficult to design new treatment strategies. The shift between different cellular substates in the same tumor context can also lead to data misinterpretation, especially when the expression of markers is associated with specific microenvironments. Assays where the expression of a marker is evaluated in a certain region of the tumor could represent only a “screenshot” of a cell state that is part of various phenotypic transitions within that same niche.

**Figure 1:**
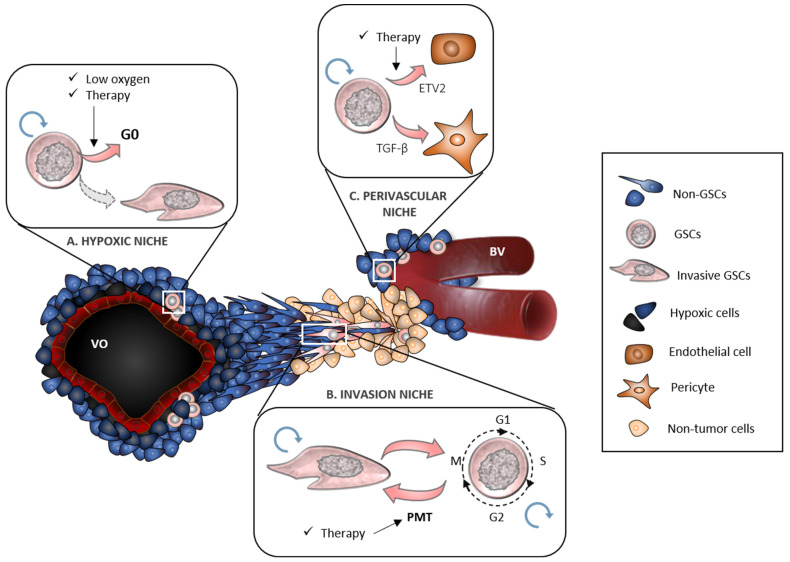
GSCs niches in the regulation of cell plasticity. GSCs persist in three different tumor niches, hypoxic, invasive and perivascular, which ensure their maintenance and self-renewal (blue arrows). (**A**). Hypoxic niche promotes GSCs quiescence, a state characterized by a reversible G0 arrest which can be induced by therapy as a resistance adaptation mechanism. Hypoxic GSCs also express migration/invasion-related proteins, thereby promoting GBM infiltration. (**B**). Invasion niche induces a proneural-mesenchymal transition (PMT) characterized by the overexpression of proteins involved in epithelial–mesenchymal transition. PMT can be induced by therapy, so it has been related with GBM recurrence. Invasion edge is composed by GSCs with high potential for extracellular matrix remodeling and colonization of healthy tissue; however, an alternate switch between migration and proliferation is essential to allow cell heterogeneity maintenance and new tumor foci formation. (**C**). Perivascular niche is composed by GSCs with the ability to remodel tumor vasculature, in part, by transdifferentiation to endothelial cells and pericytes through a mechanism regulated by ETV2 and TGF-β, respectively. This process can be induced by therapy. VO: vascular occlusion, BV: blood vessel.

Later studies focused on investigating those microenvironmental factors that promote the maintenance of the GSCs phenotype. Thus, targeting of the GSCs niches emerged as a novel therapeutic strategy since, in theory, the elimination of their biological support would decrease the pool of GSCs and their progeny. For example, Bevacizumab was proposed as a drug that would counteract GBM angiogenesis and consequently its growth. Studies that evaluated its efficacy showed that GBM could not only escape this type of therapy but also acquire a more invasive phenotype. Despite how discouraging it may seem, this background helped us to understand that each tumor niche has the ability to fill the lack of another niche thanks to the expansion of highly adapted GSCs able to reshape the microenvironment. We now know that cellular plasticity is a key property for GSCs, as it helps them persist in different niches, ensuring heterogeneity within the tumor (Figure 1). At the molecular level, this phenotypic flexibility is driven by epigenetic modifications that dynamically regulate stemness, cell fate and resistance to treatments. Since these alterations are reversible in principle, proteins of the epigenetic machinery such as DNMTs, KDMs or histone deacetylases have emerged as promising therapeutic targets. Instead of directing treatments toward specific resistance mechanisms, to components that promote niche maintenance or to signaling pathways characteristic of each molecular subtype, future therapeutic efforts could aim to counteract the plasticity of GSCs, being a strategy that would integrate all others. In other words, the manipulation of those molecular components that modulate the epigenetic landscape and their reorganization could help us enrich cell phenotypes that are more susceptible to treatments, avoiding niche remodeling and transitions toward more aggressive GSC phenotypes.

## Data Availability

Not applicable.
